# Supplementary material for: Improving uptake of prevention of mother-to-child HIV transmission services in Benue State, Nigeria through a faith-based congregational strategy
Source: PLoS One. 2021 Dec 2;16(12):e0260694. doi: 10.1371/journal.pone.0260694 (PMC8638953; doi:10.1371/journal.pone.0260694)
Supplement: S1 Questionnaires — (PDF) [file pone.0260694.s002.pdf]

Bio Data Form

Member ID #

A2 - Completed at Baby Shower Registration

Site ID#........................

....

PARTICIPANT INFORMATION

1- Surname **2 - First Name** **3- Middle Name**

4 - Age **5 - Gender** ❑ Male ❑ Female

6 - Marital Status ❑ Single ❑ Married ❑ Divorced ❑ Separated ❑ Widowed

Partner/Spouse's name:

7 - Descriptive Address

8 - Name of church you attend:

9 - Phone #1 **10 - Phone #2** **11 -Phone #3**

12 - Distance to closest health facility ❑ 0-5 km *(walk)* ❑ 6-10 km *(bike)* ❑ 11-15 km *(short ride)* ❑ >15 km *(long ride)*

13 - Occupation ❑ Farmer ❑ Trader ❑ Civil Servant ❑ Applicant ❑ Other (specify)

14-Language(s) ❑ Tiv ❑ English ❑ Igbo ❑ Hausa ❑ Yoruba ❑ Other (specify)

15-Highest level of education ❑ No education ❑ Completed Primary School ❑ Completed Junior Secondary ❑ Completed Senior Secondary ❑ Some Post-secondary ❑ Completed Post-secondary

16 - Income per month ❑ ₦0 - 20,000 ❑ ₦20,001 - 50,000 ❑ ₦50,001 - 100,000 ❑ Above ₦100,001

17-How many other people are in your household? Adults: (over 18 years): Children (under 18):

Who do you want us to call if we can't find you? Remember, we will not share information with anyone other than you.

CONTACT PERSON 1

Surname **First Name**

Relationship to participant **Phone #1**

Descriptive Address **Phone #2**

CONTACT PERSON 2

Surname **First Name**

Relationship to participant **Phone #1**

Descriptive Address **Phone #2**

Date of Baby Shower..

Completed by...Date .... Sign...

Male Health Questionnaire

Completed at Baby Shower

Member ID # ....

Vital Signs Measurements	Height:	Weight:	Blood Pressure:

Medical History

1. Has a doctor ever told you that you have hypertension? ☐ YES ☐ NO
2. Has a doctor ever told you that you have diabetes? ☐ YES ☐ NO
3. Have you had any surgeries or operations? ☐ YES ☐ NO
4. What is your genotype? ☐ AA ☐ AS ☐ AC ☐ SC ☐ SS ☐ Don't Know
5. Have you ever been tested for HIV? ☐ YES ☐ NO

5a. When was your most recent HIV test? DATE: ☐ NA

5b. What was the result? ☐ NEGATIVE ☐ POSITIVE ☐ Not Sure Don't Know 5c. On HIV Drugs? ☐ YES ☐ NO

6. Have you ever been tested for Hepatitis B? ☐ YES ☐ NO

6a. When was your most recent Hepatitis B test? DATE: ☐ NA

6b. What was the result? ☐ NEGATIVE ☐ POSITIVE ☐ Not Sure Don't Know

Lifestyle Habits

7. How often do you drink alcohol? ☐ Never ☐ Daily ☐ Weekly ☐ Monthly ☐ Occasionally

7a. When was the last time you had a drink of alcohol?
☐ Yesterday ☐ Last 1 Week ☐ Last_______ Months ☐ More than a Year Ago ☐ N/A

8. How often do you use tobacco? ☐ Never ☐ Daily ☐ Weekly ☐ Monthly ☐ Occasionally

8a. When was the last time you used tobacco?
☐ Yesterday ☐ Last 1 Week ☐ Last_______ Months ☐ More than a Year Ago ☐ N/A

9. Do you use any other substance? ☐ YES ☐ NO
If yes, specify:

9a. How often do you use this substance? ☐ N/A ☐ Daily ☐ Weekly ☐ Monthly ☐ Occasionally

9b. When was the last time you used _____________?
☐ Yesterday ☐ Last 1 Week ☐ Last_______ Months ☐ More than a Year Ago ☐ N/A

Reproductive Health

11. How many children do you have? Male: Female:
12. How many children do you want to have? Male: Female:
13. Once you have a complete family, would you support you wife in tying her womb? ☐ YES ☐ NO
14. Would you support your wife to give your baby only breast milk for at least 6 months? ☐ YES ☐ NO

Completed by:	Date:	Signature:
Reviewed by:	Date:	Signature:

Female Health Questionnaire

Completed at Baby Shower

Member ID # ....

Vital Signs Measurements	Height:	Weight:	Blood Pressure:

Medical History

1. Has a doctor ever told you that you have hypertension? ☐ YES ☐ NO
2. Has a doctor ever told you that you have diabetes? ☐ YES ☐ NO
3. Have you had any surgeries or operations or C-sections? ☐ YES ☐ NO
4. What is your genotype? ☐ AA ☐ AS ☐ AC ☐ SC ☐ SS ☐ Don't know
5. Have you ever been tested for HIV? ☐ YES ☐ NO

5a. When was your most recent HIV test? DATE: ☐ NA

5b. What was the result? ☐ NEGATIVE ☐ POSITIVE ☐ Not Sure Don't Know 5c. On HIV Drugs? ☐ YES ☐ NO

6. Have you ever been tested for Hepatitis B? ☐ YES ☐ NO

6a. When was your most recent Hepatitis B test? DATE: ☐ NA

6b. What was the result? ☐ NEGATIVE ☐ POSITIVE ☐ Not Sure Don't Know

Lifestyle Habits

7. How often do you drink alcohol? ☐ Never ☐ Daily ☐ Weekly ☐ Monthly ☐ Occasionally

7a. When was the last time you had a drink of alcohol?
☐ Yesterday ☐ Last 1 Week ☐ Last_______ Months ☐ More than a Year Ago ☐ N/A

8. How often do you use tobacco? ☐ Never ☐ Daily ☐ Weekly ☐ Monthly ☐ Occasionally

8a. When was the last time you used tobacco?
☐ Yesterday ☐ Last 1 Week ☐ Last_______ Months ☐ More than a Year Ago ☐ N/A

9. Do you use any other substance? ☐ YES ☐ NO
If yes, specify:

9a. How often do you use this substance? ☐ N/A ☐ Daily ☐ Weekly ☐ Monthly ☐ Occasionally

9b. When was the last time you used this substance?
☐ Yesterday ☐ Last 1 Week ☐ Last_______ Months ☐ More than a Year Ago ☐ N/A

Reproductive Health

10. How many months is your pregnancy? ☐ 1 ☐ 2 ☐ 3 ☐ 4 ☐ 5 ☐ 6 ☐ 7 ☐ 8 ☐ 9 ☐ Don't know
11. How many times have you been pregnant before? ☐ 0 ☐ 1 ☐ 2 ☐ 3 ☐ 4 ☐ 5 ☐ 6 ☐ 7 ☐ 8 ☐ 9 ☐ >9
12. How many children do you have? MALE: FEMALE:
13. Are you receiving antenatal care? ☐ YES ☐ NO

13a. What is the name of the health facility? NAME:

14. What is your EDD (day or month)? DATE:
15. How many children do you wish to have? MALE: FEMALE:
16. Once you have a complete family, would you consider tying your womb? ☐ YES ☐ NO
17. Did you give breast milk to your last baby? ☐ YES ☐ NO ☐ N/A (1st Baby)
18. How do you plan to feed your baby for the first 6 months? ☐ Breast milk ☐ Water ☐ Formula ☐ Pap

Completed by:	Date:	Signature:
Reviewed by:	Date:	Signature:

Modified Client Intake Form: Clinical TB and HIV Screening

Member ID

**Adapted from CLIENT INTAKE FORM (**Transfer to HCT Register Along with biodata		
**Ask patient….Have you recently had:**	YES	NO
1. Coughing for more than 2 weeks	☐	☐
2. Weight loss of equal to or more than 3 kg in the last 4 weeks	☐	☐
3. Swelling in the lymph nodes	☐	☐
4. Fever for more than 2 weeks	☐	☐
5. Night sweats for more than 2 weeks	☐	☐
6. Vaginal discharge or burning when urinating?	☐	☐
7. Lower abdominal or (for male scrotal pain)	☐	☐
8. Genital sores or swollen lymph nodes in pelvic region with or without pain	☐	☐

Last updated 07.23.16
